# Ultrasonography of Testicular Maturation and Correlation with Body Growth and Semen Evaluation in Beagle Dog Model

**DOI:** 10.3390/vetsci11060270

**Published:** 2024-06-14

**Authors:** Athina P. Venianaki, Mariana S. Barbagianni, George C. Fthenakis, Apostolos D. Galatos, Pagona G. Gouletsou

**Affiliations:** 1 Clinic of Obstetrics and Reproduction, Faculty of Veterinary Science, School of Health Sciences, University of Thessaly, Trikalon 224, 43100 Karditsa, Greece; ath.veni@gmail.com (A.P.V.); gcf@uth.gr (G.C.F.); 2 Clinic of Surgery, Faculty of Veterinary Science, School of Health Sciences, University of Thessaly, 43100 Karditsa, Greece; mabarbag@uth.gr (M.S.B.); agalatos@vet.uth.gr (A.D.G.)

**Keywords:** dog, testis, puberty, maturity, semen, ultrasound, echogenicity, heterogeneity, colour Doppler, grayscale intensity

## Abstract

**Simple Summary:**

This study examined the appearance and changes of canine testes from birth to adulthood by using ultrasound. Eight Beagle dogs were monitored from 4 to 40 weeks of life. The following parameters were evaluated every 14 days: bodyweight and height, scrotal sac and testicular volume, ultrasonographically measured testicular volume, echogenicity, heterogeneity, blood-flow score, ratio of the grayscale intensity value of the testis to the capsule, and semen quality. A correlation analysis was carried out between the various measurements obtained. Fertility was achieved in the 36th week. The echogenicity of the testicular parenchyma progressively increased with age. The ratio of grayscale intensity of testicular parenchyma had values < 200 at maturity. A colour Doppler evaluation first detected blood flow in the testis in the 22nd week, and in the 36th week > 80% of the testes imaged had visible vessels. A significant correlation was found between all the evaluation methods. The findings of the study may help clinicians to detect maturity in dogs; however, their applicability in all breeds or individuals might possibly vary due to genetic, physiological, and developmental differences. In summary, the study ultrasonographically explores the testicular maturity in dogs, with the aim to improve clinical assessments and health management in these animals.

**Abstract:**

This prospective study investigated the ultrasonographic appearance of the canine testis from birth to adulthood. Eight purpose-bred laboratory Beagle-breed dogs were monitored from 4 to 40 weeks of life. The following parameters were evaluated every two weeks: bodyweight and height, scrotal and testicular volume, ultrasonographically measured testicular volume, echogenicity, heterogeneity, blood-flow score, ratio of the grayscale intensity value of the testis to the capsule, ejaculate volume, motility, viability, and number of spermatozoa. A correlation analysis was carried out between the various measurements obtained. Fertility was achieved in the 36th week of life. The echogenicity of the testicular parenchyma increased with age, and subsequently to the 30th week of life remained constant. The heterogeneity of the testicular parenchyma, as was evaluated by the standard deviation of the values of grayscale intensity of the parenchyma, also increased with age and was >19 at the onset of fertility. The ratio of grayscale intensity of testicular parenchyma had values < 200 at maturity. A colour Doppler evaluation first detected blood flow in the testis in the 22nd week. After the 32nd week, distinct signals were visible. In the 36th week, >80% of the testes imaged had visible vessels. A significant correlation was found between all the evaluation methods. The findings of the study may help clinicians detect the onset of fertility in dogs, especially when semen evaluation is not feasible; however, their applicability in all breeds or individuals might possibly vary due to genetic, physiological, and developmental differences. In summary, the study ultrasonographically explores the testicular maturity in dogs, with the aim to improve clinical assessments and health management in these animals.

## 1. Introduction

Ultrasonographic (US) imaging is a valuable diagnostic technique, widely employed in the study of the male reproductive system in humans and domestic animals [1]. It is easy to use, non-invasive, quick to perform, and less costly compared to other imaging techniques like magnetic resonance or computed tomography [2]. The external position of the testes allows for easy access for the US examination [3]. Nowadays, US examination is commonly employed for testicular evaluation in dogs during routine abdominal examination [4,5,6]. B-mode ultrasonographic examination provides comprehensive anatomical details regarding the dimensions, the configuration and the location of testes, and their internal and adjacent structures [7,8,9,10,11]. Testicular parenchyma changes from birth to adulthood and some relevant studies in humans have described these changes [12,13]. Similar studies in animals have been performed in donkeys [14], rams [15,16,17], cats [18], horses [19], bulls [20,21,22,23], and boars [24]. In dogs, there is only one study describing the changes in fertile young dogs; however, it has not been performed on the same animals throughout their puberty [25]. A further study, performed on the same dogs from birth to maturity, only assessed differences in the haemodynamic parameters of blood flow in the testicular artery [26].

Puberty is a complex process that includes a sequence of anatomical and physiological changes. As these changes do not occur concurrently, there are multiple definitions for the start of puberty [27]. Histologically, puberty has been defined when spermatozoa can be detected in the seminiferous tubules and epididymis [27]. The testis has been evaluated ultrasonographically by the evaluation of grayscale intensity [28], which has been repeatedly described to be a valuable tool for the breeding soundness evaluation in dogs [1,7,29]. The testis consists of the connective tissue framework, called stroma, and the parenchyma, which comprises the seminiferous tubules. The stroma is created by the outer tunica albuginea, from which septa extend to divide the parenchyma into lobules [3]. The septa converge centrally at the mediastinum testis, a cord of connective tissue running lengthwise through the middle of the testis [3].

The US image display contains pixels, where each one represents a specific acoustic impedance shown in various shades of grey (grayscale intensity), ranging from absolute black (0) to absolute white (255) [30]. Acoustic impedance is connected to the density of the tissue penetrated by the acoustic beam, leading to a stronger or weaker echo. Multiple studies have explored the correlation between the grayscale intensity values of testicular parenchyma and the quality of semen in domestic animals [4,22,30,31,32,33], and humans [34,35,36,37]. These studies found a correlation between alterations in testicular pixel intensity and the proportion of morphologically normal viable sperm cells.

While US examination is routinely used to evaluate the reproductive tract in dogs, a computer-based analysis of testicular echotexture from birth to adulthood has not yet been investigated in that species. The objective of this study was the validation of this technique for clinical application in dogs. Veterinary practitioners would thus be able to apply a quick and non-invasive assessment of testicular function and structure, which can be an alternative to testicular FNA in cases of failure to collect a semen sample. The study aimed to investigate correlations between objective measures of testicular echotexture, somatometric attributes, and semen production in dogs.

## 2. Materials and Methods

### 2.1. Study Design

#### 2.1.1. Animal Ethics

The research was conducted at the research facilities of the Clinic of Surgery and the Clinic of Obstetrics and Reproduction, Faculty of Veterinary Medicine, University of Thessaly. The study’s research protocol received approval from the Greek National Animal Ethics Committee (license number: 2732/12.6.2015) and the Institutional Ethics Committee of the Faculty of Veterinary Science (protocol code 14, date of approval: 16 June 2015), ensuring compliance with European Union legislation regarding the welfare of laboratory animals. Throughout the study, all legal and ethical obligations concerning humane animal treatment and the welfare of laboratory animals were diligently met.

#### 2.1.2. Animals

Eight purpose-bred laboratory male Beagle dogs were included in the study. These originated from two litters, that had the same father, each of which had four male puppies. After birth and for four months, the dogs were housed in pens with their dam. Subsequently, the animals were moved to pens where they were housed with their littermates. The puppies were fed with a Royal Canin Starter Mother and Baby Dog for the first two months of age and thereafter with a Royal Canine Puppy for medium-sized breeds and had unrestricted access to water (Royal Canine, Aimargues, Gard, France). They were routinely treated for parasites and were vaccinated according to schedule.

#### 2.1.3. Inclusion Criteria

All dogs included in the study were clinically healthy and remained so during the experimental period, with both testes in the scrotum. Before the study, the health of each dog was assessed by clinical and laboratory examinations (full haematological and blood and urine biochemical examination); the examinations were repeated at monthly intervals throughout the study period.

### 2.2. Clinical Measurements

First, the animals were weighed. Furthermore, measurements were obtained to assess the physical conformation of each animal by measuring the height at the withers, during a natural standing posture.

The scrotum was then palpated to confirm the descent of the testes and to examine each testis and the contents of the scrotal sac. During the palpation of the testes, any pain, consistency, and reduced mobility were assessed.

To evaluate scrotal sac volume, each testis was pulled downwards as far as possible by grasping the scrotum at the level of the spermatic cord. Measurements of the length, width, and height of the scrotal sac were obtained using electronic sliding callipers after stretching the scrotal skin tightly over the testis, while the animal was placed in a supine position with the hind limbs loosely extended. The scrotal sac volume was calculated by the formula of the ellipsoid (V = 0.5236 × length × width × height) [10,38]. Afterwards, the two testes were palpated transcutaneously, and the dimensions of each testis were recorded using electronic sliding callipers. Testicular volume was also calculated using the formula of the ellipsoid [10].

### 2.3. Semen Evaluation

Attempts for semen collection began at the age of three months using digital manipulation, with the presence of a female dog, the vulva of which had been smeared with pheromones from a female in heat (swabs taken from female dogs in oestrus).

The first and second fractions of semen were collected, along with the first part of the third (prostatic) fraction, and the combined volume was recorded. The third fraction was collected over a defined time of 1 min immediately following the collection of the second fraction, because the total duration of sexual excitement might vary between individual animals, as well as the age of animals, thus affecting the total volume of the third fraction produced [4].

Each sample of ejaculate was assessed for volume, sperm motility, viability, and concentration. The volume of the ejaculate was measured with a syringe. Sperm motility was estimated subjectively to the nearest 5% by diluting a drop of semen on a slide with 0.9% sodium chloride, covering it with a coverslip, and examining it at 400× magnification on a stage incubator at 37 °C. The proportion of spermatozoa showing rapid forward movement was calculated. Moreover, during the initial semen collections, the samples were also processed without any dilution, due to the low spermatozoa concentration in the ejaculates, and there was no difference between the results obtained by either method. Sperm concentration was determined by diluting the semen 1:100 and placing the diluted sample on an improved Neubauer haemocytometer. The chambers were counted, values averaged, and sperm concentration calculated by multiplying the average number of spermatozoa by 10^6^ to obtain the number of spermatozoa per millilitre [39]. Viability (proportion of live and dead spermatozoa) was assessed on eosin-nigrosin-stained smears by counting 300 spermatozoa. While assessing viability, the smear was also evaluated for morphological abnormalities of spermatozoa. The total number of spermatozoa was calculated by multiplying the concentration by the ejaculate volume [40,41]. Motility was calculated after 20 to 40 s, a nigrosine–eosin (viability) smear was made after 30 to 60 s, and the other parameters were evaluated within the following 2 to 6 h after semen collection.

### 2.4. Ultrasonographic Evaluation

The US examinations were consistently conducted after the morning feeding, starting from the 4th to the 40th week of age at two-week intervals. The examination was performed with the animal in a lateral recumbent position, with no sedation administered during the procedures. All US examinations were carried out by the same operator (A.P.V.). On average, the total duration of the procedure for all regions of the two testes was approximately 20 (min. 17–max. 26) minutes. The hair on the scrotum was fully shaved for the examination and coupling gel (Aquasonic^®^ 100, Parker, Fairfield, NJ, USA) was applied to ensure that no pressure was applied to the scrotum during the examination. Each testis was examined individually. The procedure began with the right testis while the animal was in the left lateral position; the dog was then placed in the right lateral position for the examination of the contralateral testis.

**Figure 1:**
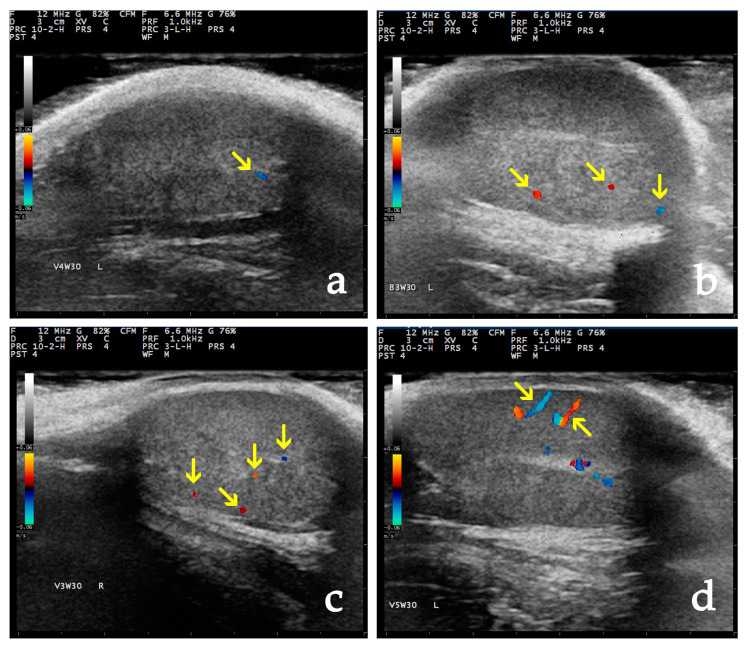
Colour Doppler scores of the testicular parenchyma vessels. (**a**). Score 1: one vessel (colour pixels) is visible (yellow arrow). (**b**). Score 2: distinct signals (three vessels) are visible (yellow arrows). (**c**). Score 3: distinct signals (four vessels) are visible (yellow arrows). (**d**). Score 4: the course of the vessels is visible (yellow arrows).

An ultrasound scanner (MyLab^®^ 30, ESAOTE, Genoa, Italy) equipped with a linear transducer operating at an imaging frequency of 7.5 to 12.0 MHz was utilized. The settings applied included a frequency of 12.0 MHz and a scanning depth of 20 to 40 mm. For the examination, the transducer was positioned initially on the neck of the scrotum, cranially to the testis, to image all tissues, and the focal point (focus) was positioned directly beneath the distal portion of the tunica albuginea. The other ultrasound settings, such as gain, brightness, and contrast, were standardized at the machine median settings. Following the B-mode examination, the colour Doppler mode was activated, with the pulse repetition frequency maintained at 1.4 kHz, and the description of the patterns of the parenchymal vessels was colour-coded and assessed with a modified version of the methodology of Gumbsch [42] as follows (Figure 1):

Score 0: no blood flow could be detected.

Score 1: one to three vessels (colour pixels) were visible.

Score 2: distinct signals (up to three vessels) were visible.

Score 3: distinct signals (over three vessels) were visible.

Score 4: the course of the vessel was visible.

### 2.5. Management of Ultrasonographic Images

The images of the testes were captured in sagittal and transverse planes, with gentle pressure to maintain the testicular shape integrity. The images were saved on a hard disk. The testicular dimensions were measured using electronic cursors on the equipment, placed at the tunica albuginea borders. Each testis was imaged in at least three separate sagittal and transverse views, with the largest measurement recorded for each dimension used for volume calculation [41]. The length, height, and width of the testes were measured to calculate the total testicular volume = l × w × h × 0.71, where l = sagittal diameter, w = transverse diameter, and h = dorsal diameter [10].

**Figure 2:**
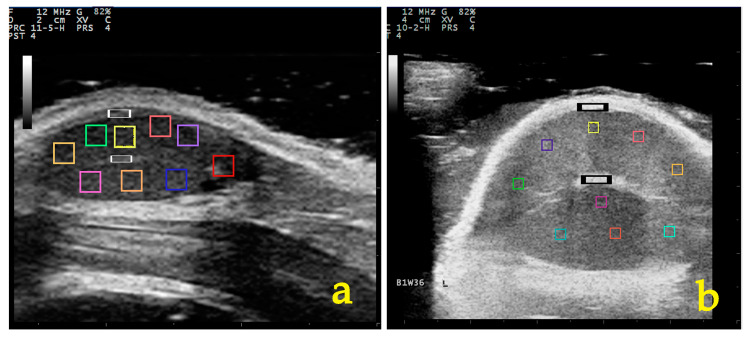
The spot-meter technique; a total of nine square-shaped spots (2 mm^2^) of testicular parenchyma are selected for evaluating grayscale intensity values (coloured squares). The grayscale intensity values of the capsule of the testis and mediastinum testis are also evaluated ((**a**): white rectangles; (**b**): black rectangles).

**Figure 3:**
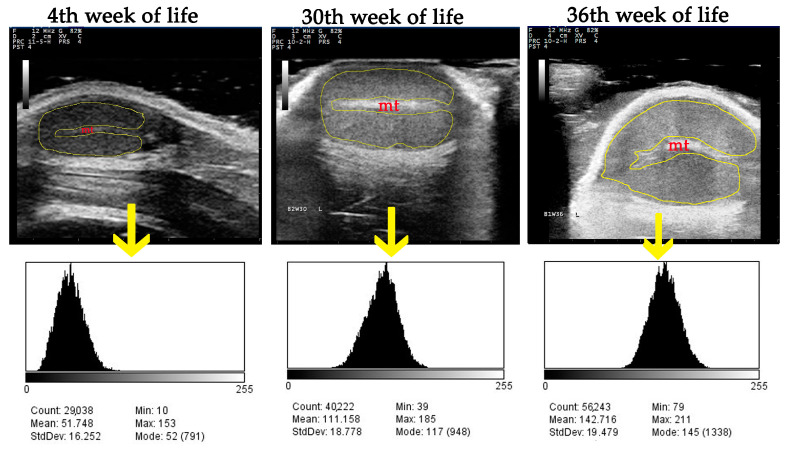
The region-area technique of grayscale analysis: the entire image of testicular parenchyma is outlined (yellow line), excluding mediastinum testis (mt), and corresponding histograms are created for the calculation of mean pixel grayscale intensity (numerical pixel values) and pixel heterogeneity (pixel values standard deviations).

A computer-assisted analysis of the US images was performed using two techniques: the spot-meter technique and the region-area technique. For both, the National Institutes of Health’s free research software (ImageJ-Fiji, NIH, Bethesda, MD, USA, 2023) was employed. For the spot-meter technique, a total of nine square-shaped spots (2 mm^2^) were selected on each saved image of testicular parenchyma, excluding any image artefacts from the measurements (Figure 2). For the region-area technique, an entire image of testicular parenchyma was outlined using a free-form tracing tool, with the mediastinum testis excluded from the measurements, as described by Pozor et al. [19]. Mean pixel grayscale intensity (numerical pixel values) and pixel heterogeneity (pixel values standard deviations) were calculated for each image and each technique (Figure 3). The grayscale intensity was defined as grayscale values of individual picture elements ranging from 0 (absolute black, fully anechoic) to 255 (absolute white, fully hyperechoic). The echogenicity of the testicular parenchyma was categorised by allocating the grayscale intensity values into one of three categories: low echogenicity (0–85), moderate echogenicity (86–170), and high echogenicity (171–255), as described by da Silva Ribeiro [43].

In addition, the formula proposed by Moxon et al. [4] was used to establish a single reference point, with the aim of standardizing all testicular grayscale intensity measurements in dogs. The computerized pixel measurements of the grayscale intensity values of a highly echogenic structure, such as the tunica albuginea (or capsule) of the testis, were proposed as the reference point and the percent echogenicity of the testicular parenchyma (percent echogenicity = mean pixel intensity of the capsule/mean pixel intensity of the testicular parenchyma × 100) was calculated. In the present study, a modification of the above formula was also used and tested. Here, the grayscale intensity of the testicular mediastinum was used to calculate the percent echogenicity of the testicular parenchyma, i.e., percent echogenicity = mean pixel intensity of the testicular mediastinum/mean pixel intensity of the testicular parenchyma × 100.

The following parameters were calculated: testicular parenchyma grayscale intensity values (spot-meter technique and region-area technique), testicular heterogeneity (standard deviation of grayscale intensity values), mediastinum testis grayscale intensity values, testis capsule grayscale intensity values, the ratio of the grayscale intensity values of the mediastinum testis to the grayscale intensity values of testicular parenchyma (ratio mediastinum testis/parenchyma), and the ratio of the grayscale intensity values of the capsule of the testis to the grayscale intensity values of testicular parenchyma (ratio capsule/parenchyma).

### 2.6. Data Management and Analysis

Data were entered into Microsoft Excel (Microsoft Corporation, Redmond, Washington, USA) and analysed using SPSS v. 21 (IBM Analytics, Armonk, New York, NY, USA, 2020). A basic descriptive analysis was performed.

To study changes over time, the period of the experimental study was divided into three age-related periods. The periods were chosen to reflect the different phases of testicular maturation and sperm production: the first from birth to the first ejaculation (weeks 4–28, “pre-pubertal period”), the second from the first ejaculation to the first recording of spermatozoa in the ejaculate (weeks 30–34, “pubertal period”), and the third subsequently to the first recording of fertile numbers of spermatozoa in the ejaculate (>200 million) until the end of the study (weeks 36–40, “post-pubertal period”).

Initially, all values related to the various parameters assessed at individual testis levels were compared between the left and the right testis for all animals. As no significant differences were found between the left and right testis of all animals at all examination points (*p* > 0.60 for all comparisons), these parameters were then considered together for subsequent analyses.

On each testis, separate grayscale intensity values were calculated for the testicular parenchyma, the capsule of the testis and the mediastinum testis. Differences in grayscale intensity values between these three anatomical structures were assessed by using the Kruskal–Wallis test.

For each testis and examination day, the results of grayscale intensity obtained by each of the two techniques employed, the spot-meter technique and region-area technique, were compared by using Spearman’s rank correlation. Finally, correlation analysis, also by using Spearman’s rank correlation, was performed between all the parameters calculated during the study, by taking into account all values obtained from all animals at all examination points.

Statistical significance was defined at *p* < 0.05.

## 3. Results

### 3.1. Clinical Measurements of Animal Growth

The median, minimum, and maximum weight and height of the animals are presented in Figures S1 and S2 and Table S1.

### 3.2. Clinical Examination of the Scrotum

The palpation of the scrotum indicated symmetry between the right and left testis, physiological consistency and thin scrotal skin. Up to the end of the study, no animal was observed with signs of pain, swelling of the scrotum, clinically detected inflammation, or restricted movement of testes into the vaginal cavity.

**Figure 4:**
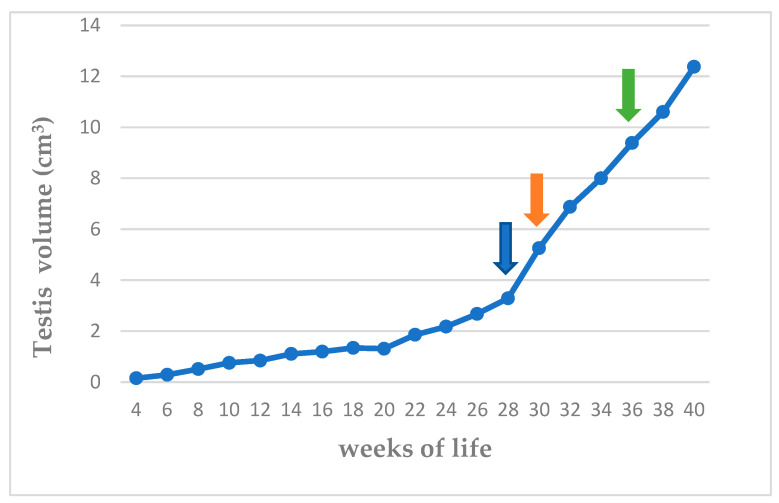
Median testicular volume (cm^3^) from 4th to 40th week of life (blue arrow: first ejaculate; orange arrow: spermatozoa first seen in ejaculate; green arrow: total sperm count > 200 × 10^6^ in ejaculate).

The clinically evaluated scrotal volume is presented in Figure S3 and Table S1 and the clinically evaluated testicular volume in Figure 4 and Table S1. No difference was found between the volume of the right and the left testis (*p* > 0.60). The volume of the testes continued to increase after the attainment of fertility (36th week of life).

### 3.3. Semen Evaluation

**Figure 5:**
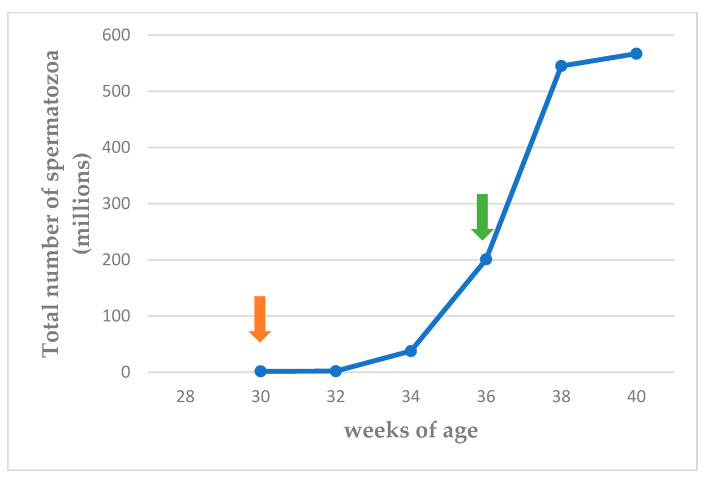
Median total number of spermatozoa (×10^6^) from 30th to 40th week of life (orange arrow: spermatozoa first seen in ejaculate; green arrow: total sperm count > 200 × 10^6^ in ejaculate).

Ejaculates could be first collected from most animals in the 28th week of life; in two semen samples, some spermatozoa were detected after a thorough examination of the samples; however, no other evaluation was feasible. After two weeks, in the 30th week of life, spermatozoa were detected in all semen samples; however, in some of them, their number was extremely low. The spermatozoa did not show rapid forward movement at that time; however, two weeks later, motility was 50 to 80%. Viability, as determined with nigrosine–eosin stain, was over 80% in all evaluated samples. While evaluating viability in nigrosine–eosin-stained smears, no primary morphological abnormalities were seen; the only secondary abnormality observed, in <5% of spermatozoa, was coiled tails, probably a result of the handling of the sample. However, it is noted that in some samples fewer than 300 spermatozoa could be evaluated due to their very small number in the respective sample. Fertile semen samples (total sperm count > 200 × 10^6^) were collected from the 36th week of life onwards in the majority of the animals. The volume of the ejaculate and the sperm motility and viability are presented in Table S2 and Figures S4 and S5. The total number of spermatozoa is presented in Table S2 and Figure 5.

### 3.4. Ultrasonographic Examination

**Figure 6:**
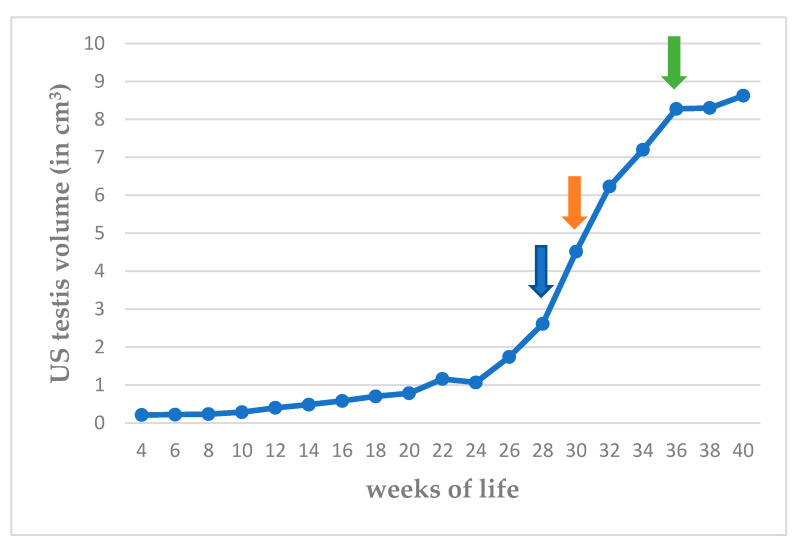
Median ultrasonographically (US) evaluated testicular volume (cm^3^) from 4th to 40th week of life (blue arrow: first ejaculate; orange arrow: spermatozoa first seen in ejaculate; green arrow: total sperm count > 200 × 10^6^ in ejaculate).

During the B-mode US examination, it was feasible to identify and image the testes within the scrotum, from the 4th week of life in all the animals in the study. The testes were imaged as oval-shaped formations during longitudinal and transverse sections, the size of which was seen to increase progressively, slowly until the 24th week and rapidly thereafter until the 36th week of life. The median values of ultrasonographically evaluated testicular volume are presented in Figure 6 and median, minimum, and maximum values are presented in Table S3.

The mediastinum testis was imaged clearly in one animal in the 4th week, in 6 animals in the 6th, and in all animals in the 8th week of life.

**Figure 7:**
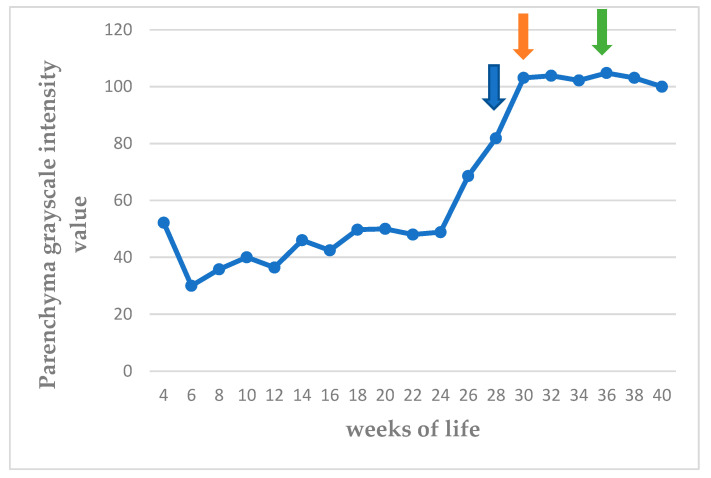
Median grayscale intensity values (scale 0–255) of testicular parenchyma, from 4th to 40th week of life (blue arrow: first ejaculate; orange arrow: spermatozoa first seen in ejaculate; green arrow: total sperm count > 200 × 10^6^ in ejaculate).

**Figure 8:**
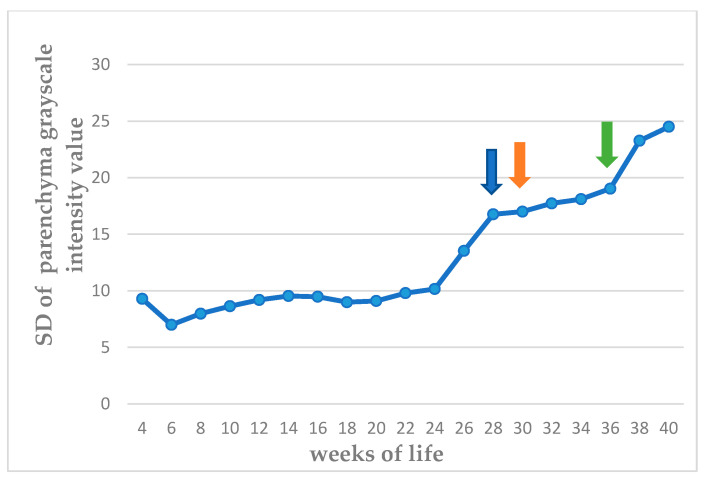
Median of standard deviation (SD) of grayscale intensity values of testicular parenchyma, from 4th to 40th week of life (blue arrow: first ejaculate; orange arrow: spermatozoa first seen in ejaculate; green arrow: total sperm count > 200 × 10^6^ in ejaculate).

**Figure 9:**
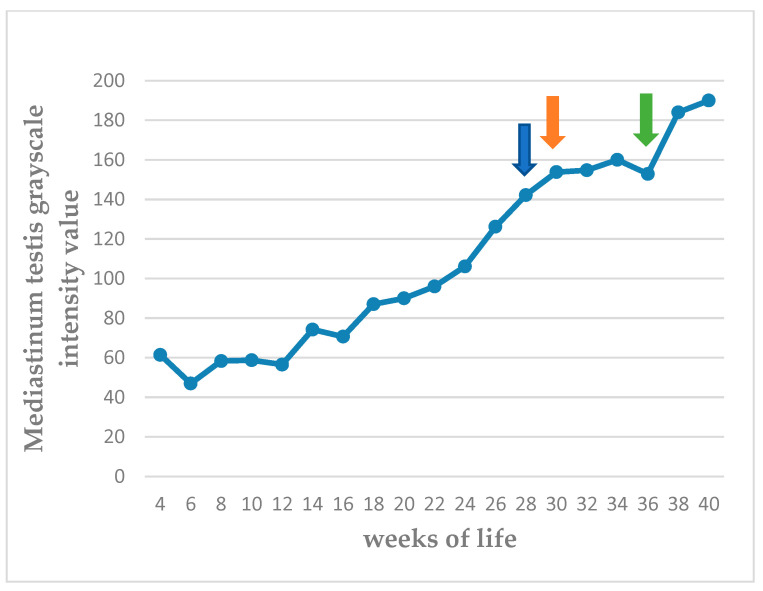
Median grayscale intensity values (scale 0–255) of mediastinum testis from 4th to 40th week of life (blue arrow: first ejaculate; orange arrow: spermatozoa first seen in ejaculate; green arrow: total sperm count > 200 × 10^6^ in ejaculate).

**Figure 10:**
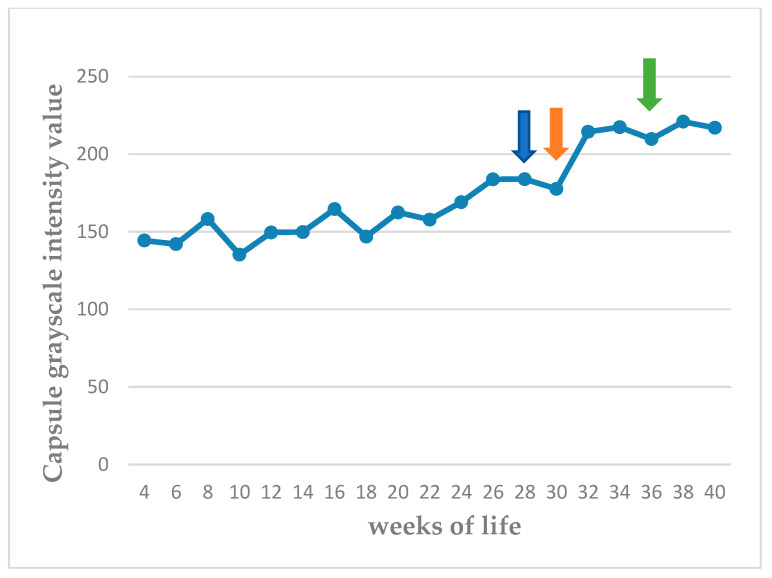
Median grayscale intensity values (scale 0–255) of tunica albuginea (testis capsule) from 4th week to 40th week of life (blue arrow: first ejaculate; orange arrow: spermatozoa first seen in ejaculate; green arrow: total sperm count > 200 × 10^6^ in ejaculate).

The echogenicity of the parenchyma of the testes progressively increased as the puppies grew, from hypoechoic to medium echogenicity. The grayscale intensity values of the testicular parenchyma, the capsule of the testis, and the mediastinum testis also increased significantly as the puppies grew (*p* < 0.0001). The slopes of the changes in grayscale intensity values were 2.525 ± 0.289 for the parenchyma and 4.251 ± 0.301 for the mediastinum testis (*p* < 0.0001 for the progressive changes). There was also a clear difference in the rate of change between the two measurements (*p* = 0.0002). The standard deviation of the grey intensity values of the testicular parenchyma, which reflects the heterogeneity of the testis, also increased significantly with age (*p* < 0.0001). The values of grey intensity of the aforementioned areas are presented in Figure 7, Figure 8, Figure 9 and Figure 10 and Tables S3 and S4.

**Figure 11:**
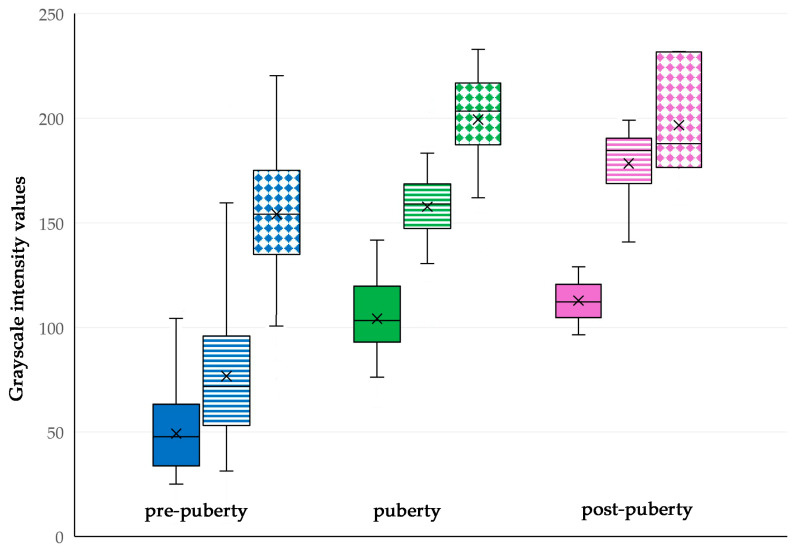
Box and whisker plot of grayscale intensity values (scale 0–255) during pre-pubertal (blue), pubertal (green), and post-pubertal (violet) period (solid blocks: testicular parenchyma; lined blocks: mediastinum testis; rhombus mesh: testis capsule) (*p* < 0.0001 for all comparisons).

There was significant difference between the median values of grayscale intensity of the testicular parenchyma, mediastinum testis, and testicular capsule for the pre-pubertal (4–28 weeks of life), the pubertal (30–34 weeks of life) and the post-pubertal (36–40 weeks of life) periods between them and among each period (*p* < 0.0001 for all comparisons) (Figure 11).

**Figure 12:**
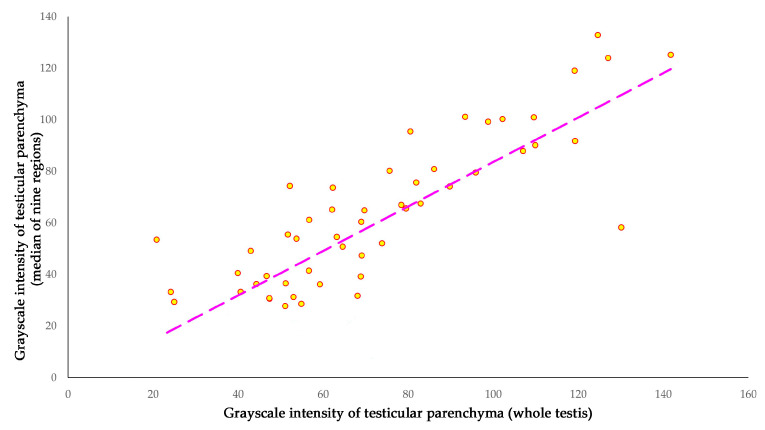
A cross-plot of grayscale intensity values (scale 0–255) of all the area of testicular parenchyma (except the mediastinum testis) and the area of separate sampling nine different regions of interest.

There was also a significant correlation between the two different techniques used for calculating the grayscale intensity of the testicular parenchyma, i.e., all the areas of testicular parenchyma (except the mediastinum testis) and the area of separate sampling nine different regions of interest (*r_sp_* = 0.751, *p* = 0.001) (Figure 12).

**Figure 13:**
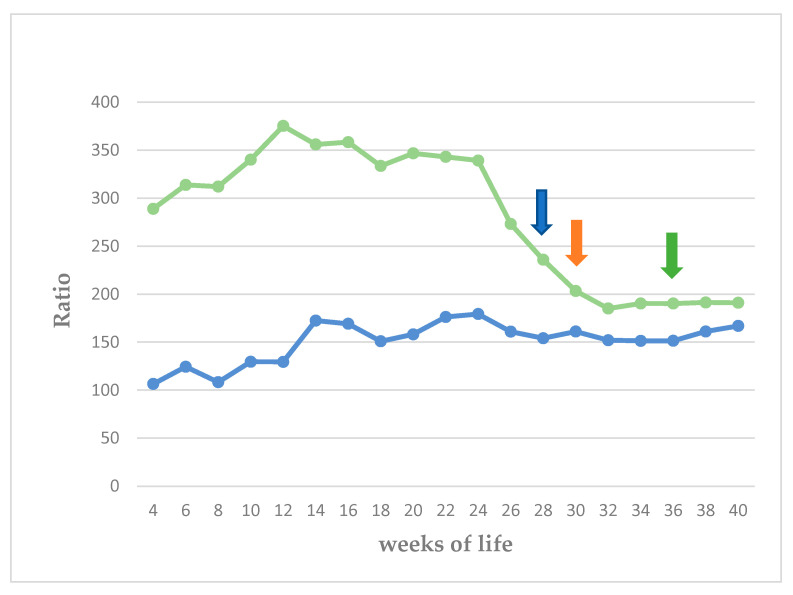
Ratio of grayscale intensity values of mediastinum testis/testicular parenchyma (blue line) and ratio of grayscale intensity values of capsule/testicular parenchyma (green line) (blue arrow: first ejaculate; orange arrow: spermatozoa first seen in ejaculate; green arrow: total sperm count > 200 × 10^6^ in ejaculate).

There was a significant difference in the progressive change in the ratios of grayscale intensity values of testicular capsule to testicular parenchyma and grayscale intensity values of mediastinum testis to testicular parenchyma (*p* = 0.0001 and *p* = 0.011, respectively) (Figure 13). However, there was a difference between the three periods only for the ratio of testicular capsule to testicular parenchyma (*p* = 0.0001).

### 3.5. Colour Doppler Evaluation

The proportion of testes scored with each of the five possible colour Doppler scores throughout the study is presented in Table 1. Up to the 20th week of life, all testes were assessed with a score of zero. Thereafter, the score increased gradually and after the 32nd week of life, all testes were evaluated with scores of three or four.

### 3.6. Correlations

The correlation analysis showed mostly significant positive correlations between all parameters assessed (*r_sp_* > 0.685, *p* < 0.001 for all calculations). Notable exceptions were (a) assessments for the ratio of grayscale intensity values of testicular capsule to testicular parenchyma, where significant inverse correlations were seen (*r_sp_* < −0.710, *p* < 0.001 for all calculations), and (b) assessments for the ratio of grayscale intensity values of mediastinum testis to testicular parenchyma, where some of these did not yield significant results (*r_sp_* < 0.475 for all calculations) (Table 2 and Table 3).

**Figure 14:**
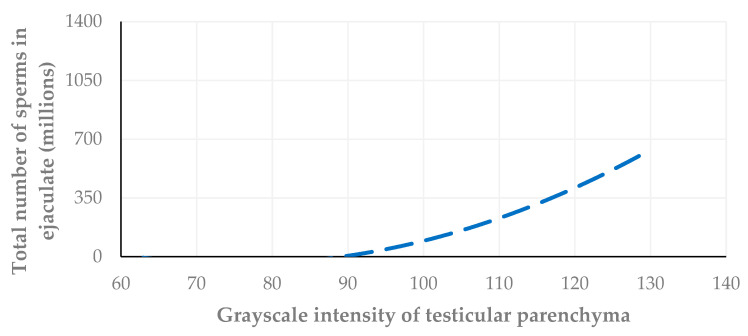
A cross-plot of the grayscale intensity values of testicular parenchyma and the total number of spermatozoa in the ejaculate collected the same day with the US examination (*r_sp_* = 0.726, *p* = 0.001).

**Figure 15:**
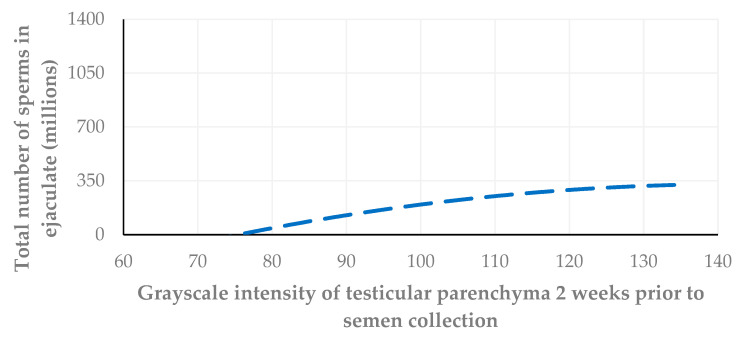
A cross-plot of the grayscale intensity values of testicular parenchyma and the total number of spermatozoa in the ejaculate two weeks after the US examination (*r_sp_* = 0.680, *p* = 0.003).

**Figure 16:**
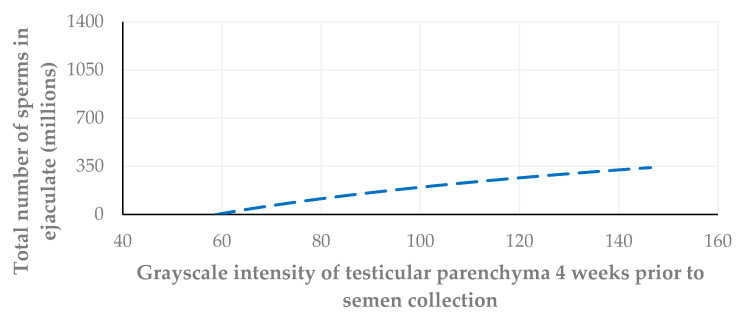
A cross-plot of the grayscale intensity values of testicular parenchyma and the total number of spermatozoa in the ejaculate four weeks after the US examination (*r_sp_* = 0.617, *p* = 0.008).

Further, the grayscale intensity values of testicular parenchyma were correlated with the total number of spermatozoa in the ejaculate collected on the same day, as well as two weeks and four weeks after the US examination. The highest correlation was found between the grayscale intensity values of testicular parenchyma and the spermatozoa number in the sample collected on the day of the US examination (*r_sp_* = 0.726, *p* = 0.001, *r_sp_* = 0.680, *p* = 0.003; *r_sp_* = 0.617, *p* = 0.008) (Figure 14, Figure 15 and Figure 16).

## 4. Discussion

### 4.1. Clinical Measurements of Animals’ Growth

Body weight has been found to have a highly positive correlation with testicular volume, which, in turn, is correlated to sperm production [4,44,45]. In our study, the weight and height of the animals had reached, respectively, 86% (83–92%) and 95% (88–96%) of the maturity values when the first spermatozoa were found in the ejaculate; thereafter, they had reached 95% (92–100%) and 99% (87–100%) of maturity values, respectively, when a fertile number of spermatozoa was recorded in the ejaculate.

### 4.2. Clinical Examination of the Scrotum

Testicular weight has been associated with testicular size through different testicular measurement techniques, for example, direct measurement of the total scrotal width [46] or measurement of the length and width of the testes through the scrotum [45]. Other techniques that have been used for estimating testicular dimensions or volume include the use of Prader [47] or Rochester [48] orchidometers, the use of rulers or callipers [49,50], and US examination [8,9,10,51].

Determining the testicular volume is important for assessing pubertal development. Testicular volume largely reflects spermatogenesis [52], as approximately 70–80% of testicular mass consists of seminiferous tubules. Testicular volume has been repeatedly associated with total sperm count, sperm motility, sperm morphology, and daily sperm production [27,46,48,53,54,55,56]. In previous studies, a direct relationship between testicular size and sperm production has been established, in various animal species, e.g., bulls [57], stallions [58], and male dogs [27,46]. Mialot et al. [27] found that the testicular growth pattern in dogs was sigmoid, with the maximal growth occurring between 22 and 36 weeks, just after the period of maximal body weight gain. In the present study, the maximal growth occurred between the 24th and 40th week of life. Gouletsou et al. [10], who clinically measured the testicular volume in 21 mature Beagle dogs, found a mean of 13.9 cm^3^, and in another research [41] a mean of 12.8 cm^3^, with both findings being in accordance with those in the present study.

As sperm output in dogs is linked to testicular size, an accurate assessment of testicular size can assist clinicians in determining whether a dog is efficiently producing and ejaculating an optimal number of spermatozoa.

In the present study, the clinically evaluated scrotal sac volume had reached 61% (57–90%) of the maturity volume and the clinically evaluated testicular volume had reached 42% (34–78%) of the maturity volume when spermatozoa were first seen in the ejaculate. Scrotal volume had reached 95% (91–97%) and testicular volume had reached 75% (95–100%) of the maturity volume, when fertile numbers of spermatozoa were also recorded.

### 4.3. Semen Evaluation

The viability (percentage of live spermatozoa) in our study was over 80% (70–100%) in the samples with fertile numbers of spermatozoa. At the 30th week of life, the small number of spermatozoa in the ejaculate did not show forward movement; however, two weeks later, motility varied from 50% to 80%. According to Gobello [59], the first ejaculations from dogs after puberty often have a high number of dead or abnormal spermatozoa, which suggests that spermatogenesis continues to develop during the period around puberty. In the present study, we also found no forward movement of spermatozoa in the first ejaculations, but no abnormal spermatozoa were seen in the ejaculates.

Mialot et al. [27] reported that the first spermatozoa in the ejaculate were seen at the end of the period of rapid testicular growth, between the 32nd and 34th week of life, whilst the total number of spermatozoa per ejaculate showed a rapid increase after the 40th week; values over 250 × 10^6^ spermatozoa were found at the 44th week. Those authors also found that the proportion of dead spermatozoa was high during the first weeks (25–50%), but then decreased to below 20% after the 40th week, whilst motility increased after that.

Gouletsou et al. [41], who performed serial semen examinations in 18 sexually mature (1–2 years old) healthy Beagles found that the mean volume of ejaculate was 1.87 mL, the mean proportion of motile sperms 89.4% (>80% in most samples), and the mean total spermatozoa number was 363 × 10^6^ sperms, thus confirming that in the present study, the onset of maturity was achieved after the 36th week of life.

### 4.4. Ultrasonographic Examination

#### 4.4.1. Testicular Volume

An US examination has been used to estimate testicular size in vivo in dogs [8,9,10,41,51,60] and was found to accurately measure the dimensions and volume of testicular parenchyma [10]. According to Evans et al. [3], the typical size dimensions of a mature testis are around 3 cm (major axis) to 2 cm (minor axis) in an 11 kg dog. However, the significant variation in canine body weight across breeds leads to a wide range of testicular sizes within this species [1]. For the Beagle breed, Gouletsou et al. [10] found a mean volume of 9.5 cm^3^, whilst in a later study the same research group [41] reported a mean testicular volume of 8.3 cm^3^, with both findings in concordance with those of the present study. In another study of testicular size in large-breed dogs, de Souza et al. [60] found that the testicular volume was significantly larger in post-pubertal (11.5  ±  2.1 cm^3^) compared to pre-pubertal (4.3  ±  3.2 cm^3^) dogs. In mature large-breed dogs, Moxon et al. [4] found no relationship between the age and any of the US measurements of testes, whilst Mantziaras et al. [61] found a tendency for testicular volume to increase during maturity until approximately 6 years of age and then to decrease.

In the present study, during the B-mode US examination, the testes were identified and imaged within the scrotum from the 4th week of life in all the puppies in the study; however, their size was very small (0.2 cm^3^). This increased slowly until the 24th week of life and rapidly thereafter and until the 36th week, when fertility was achieved, with the testes reaching 96% (72–98%) of the maturity size. The cessation of the increase in testicular size in repeated measurements might be used to identify maturity and the onset of fertility.

#### 4.4.2. Testicular Echogenicity

The computer-based analysis of echotexture is an objective method to evaluate testicular echogenicity and heterogeneity, an alternative to invasive techniques of assessing male reproductive functions [4,19,32]. This analytical method is based on noting changes in pixel grayscale intensity values and pixel allocation within the US images of testicular parenchyma that are demonstrably related to microstructural attributes of the testes [30,31,62]. Pozor et al. [19] compared two different methods of sampling US images of testicular parenchyma in stallions for pixel analysis, the spot-meter technique and the region-area technique, and found no differences in testicular parenchyma grayscale intensity values between them. However, the standard deviation of the grayscale intensity values was significantly higher when obtained with the region-area technique, probably because the mediastinum testis was included within the area analysed. In the present study, we also assessed both these techniques; however, we excluded the mediastinum testis area in the region-area technique to avoid a potential bias in the results. For the spot-meter technique, we followed the methodology performed on dogs by Moxon et al. [4] and England et al. [32], who assessed nine sampling regions of interest within each testis. In the present study, the two techniques showed a statistically significant positive correlation, indicating that either method can be used, at least when the testicular parenchyma examined does not have focal lesions, as it was in our study on the maturation of normal testes. However, in young dogs, it might be more difficult to apply the spot-meter technique proposed by Moxon et al. [4], because it is based on evaluating the regions of a standard area that might not fit within the small testes.

Various studies have been performed on the testicular echogenicity of bulls, rams, horses, dogs, and men, indicating an increase during sexual development [3,12,22,63,64,65] when the most active phase of the seminiferous tubules’ growth occurs [19]. In men, the observed increase in testicular echogenicity during puberty has primarily been explained by the morphologic changes that occur in testicular parenchyma during maturation [12]. The human testis goes through various developmental stages from birth to maturity: a resting phase, from birth until the age of approximately 4 years, a phase of active growth, between 4 and 9 years, and a maturation phase, from the age of 9 years to adulthood [66,67]. The most striking testicular changes after birth occur during puberty when the seminiferous tubules become elongated and twisted, increase in diameter and form a lumen, whilst the basement membrane becomes thicker [66,68,69]. Although these histological findings cannot fully explain the changes in testicular echogenicity, which appear to be more complex, it is clear that testicular echogenicity correlates with the maturation of the testis [12].

In the present study in dogs, the echogenicity of the testicular parenchyma slightly decreased from 4 to 6 weeks of life, a finding that is in concordance with a similar study in infants, where Hamm and Fobbe [12] noticed a slight decrease in echogenicity during the first 2 years of life. The echogenicity of the testicular parenchyma in the present study progressively increased with age, from being hypoechoic to having medium echogenicity. Likewise in kids, testicular echogenicity increased at the age of 9 years, with the values becoming especially pronounced between the ages of 12 and 15 years, i.e., during puberty. In the 30th week of life of the young dogs, when the first spermatozoa were seen in the ejaculate, grayscale intensity values had reached a plateau and remained stable afterwards. Similarly, in humans, after the completion of puberty, no further change was found to occur as shown by the comparison of the 16- and 30-year-old males in the study of Hamm and Fobbe [12], who reported no significant difference in testicular echogenicity between these two age groups. In horses, Pozor et al. [19] also found significantly lower grayscale intensity values in the testes from colts (<1 year), compared to the testes from young and mature stallions. The largest difference was observed between colts that were seven or eight months old and young stallions that were thirteen months old, indicating that the changes in testicular structure and US appearance in horses were noticed directly before puberty. Pozor et al. [19] also found no differences in the grayscale intensity of the testes in young stallions (1–5 years old) in comparison with mature stallions (>5 years old). In bulls aged 14–26 to 70–74 weeks, Brito et al. [22] found that the testicular echogenicity started to increase 16–12 weeks before puberty and reached maximum values 4 weeks before or at puberty, whilst after puberty the composition of the parenchyma remained consistent. In rams, da Silva Ribeiro et al. [43] found that the testicular parenchyma had low echogenicity in pre-pubertal animals and medium echogenicity in the pubertal and sexually mature ones. Andrade et al. [70], who also compared the testicular parenchyma of pre-pubertal and pubertal Santa Ines sheep, found a predominance of low echogenicity images in prepubertal animals and moderate echogenicity images in pubertal animals.

It seems that the grayscale intensity of testicular parenchyma follows the same pattern in relation to puberty in a variety of species, regardless of the final testis size.

### 4.5. Ultrasonographic Examination and Semen Characteristics

#### 4.5.1. Testicular Echogenicity

In a study in rams, Ahmadi et al. [31] concluded that scrotal US examination combined with computer-assisted analyses of testicular echotexture was a valuable method for determining certain current and future semen parameters. Brito et al. [22], in a study on bulls, found that testicular echogenicity was associated with sperm production and sperm morphology, but the associations were not consistent. Arteaga et al. [30] found that, in bulls with induced testicular degeneration, testicular parenchymal pixel intensity values were better associated with future semen quality than with semen quality at the time of the examination. This delay in changes in the parameters of ejaculated semen was attributed by the authors to the time required for the final stages of spermatogenesis, epididymal maturation, and transport. Interestingly, in dogs, testicular blood flow measured by the spectral Doppler technique has been related to semen quality close to the time of ultrasound examination rather than future semen quality, as would be expected [11,60]. England et al. [32], in a study in mature dogs, found that ultrasound-related parameters were not predictive of future total sperm output or percentage of live normal sperm, but mean testicular echogenicity was positively related to future sperm motility.

In the present study, when the grayscale intensity values of the testicular parenchyma were examined for a potential association with the total number of spermatozoa in the semen sample collected on the same day, a significant positive correlation was found. When grayscale intensity values were examined for their association with the total number of spermatozoa in the semen two and four weeks later, a less strong but still significant positive correlation was also evident.

#### 4.5.2. The Heterogeneity of the Testis

The heterogeneity of the testis is depicted by the standard deviation of the grayscale intensity values of the testicular parenchyma [4,19,31,32]. Moxon et al. [4] reported that, in mature dogs, mean testicular heterogeneity correlated positively with total spermatozoa, a finding that is in accordance with our results, where the increase in heterogeneity of the testes was associated with the onset of sperm production. The heterogeneity of the testis in our study increased significantly with age and was >19 (scale 0–255) when fertility was achieved, further increasing afterwards. Moxon et al. [4] measured testicular heterogeneity by examining the standard deviation of the mean pixel intensity values of nine regions of interest. These researchers proposed that a refinement of the methodology would be to sample a larger area and consider the statistical evaluation of the range of values for grayscale intensity rather than to calculate the mean value, which by nature tends to smooth the data, as was the methodological approach used in the present study. Pozor et al. [19] stated that in horses, the heterogeneity of the testicular parenchyma was lower in colts less than a year old than in older animals; however, no differences were found between different months of age within any of the age groups. In a study on rams, the heterogeneity of testicular echotexture was directly associated with biochemical components of the testicular tissue, such as extractable lipids [31].

#### 4.5.3. Other Ultrasonographic Findings

The mediastinum testis was imaged clearly in all testes in the 8th week of life and showed an increasing grayscale intensity until maturity, exceeding the value of 190 (scale 0–255), when fertility was achieved. The grayscale intensity of the capsule of the testis also showed increasing grayscale intensity; however, this increase was less prominent and reached a plateau, with values between 210 and 220, subsequently to the 32nd week of life.

When the grayscale intensity values of the three anatomical sections of the testis (parenchyma, mediastinum testis, capsule) were evaluated for the pre-pubertal, the pubertal, and the post-pubertal period, it was found that there was a significant difference between all of them.

All the aforementioned methods for evaluating the echogenicity and the heterogeneity of the testis, by using grayscale intensity values, might be useful in predicting the onset of fertility during puberty, especially in animals where semen evaluation is not possible.

One of the problems with the evaluation of the echogenicity of the testes is that it might vary, due to differences in the equipment and the various settings employed during the examination. Moxon et al. [4] proposed a formula to establish a consistent reference point in order to standardize all grayscale intensity measurements across dogs. For a reference point, the computer-based pixel measurements of grayscale intensity values of a highly echogenic structure, as the tunica albuginea (or capsule) of the testis, was proposed and the percentage echogenicity of the testicular parenchyma (percentage echogenicity = mean pixel intensity of capsule/mean pixel intensity of testicular parenchyma × 100) was calculated. In the present study, the percentage echogenicity of testicular parenchyma to the testicular capsule (ratio capsule / testicular parenchyma) was found to have significant negative correlations to all other evaluations performed, whilst values <200 were found during maturity, indicating that this value might be used for predicting the onset of fertility after puberty. In the study of Moxon et al. [4], the mean proportion of testicular echogenicity (compared to the testis capsule) was inversely correlated to the proportion of morphologically normal live sperm. However, the findings of the present study obtained from animals monitored from birth to the 40th week of life, which had no pathological conditions, and their spermatozoa output was >200 × 10^6^, cannot be correlated to the findings of Moxon et al. [4], since some of the animals in their study were of advanced age and some had low sperm output.

The mediastinum testis, another highly echogenic structure, was also tested for use as a consistent reference point, in order to standardize all grayscale intensity measurements across dogs. Nevertheless, the percentage echogenicity of the testicular parenchyma to mediastinum testis did not show significant correlations to the other parameters evaluated. Specifically, it did not change significantly with the onset of fertility, so this method cannot be used for the prediction of the onset of fertility after puberty.

#### 4.5.4. Colour Doppler of the Testes

With regard to the colour Doppler evaluation of the testes in the present study, blood flow could be first detected within the testicular parenchyma (score 1) in the 22nd week of life. Venianaki et al. [26], in a study on the haemodynamic parameters at different segments of the testicular arteries during pre-puberty, puberty, and post-puberty, also first obtained pulsed-wave Doppler measurements in the intratesticular artery in the 22nd week of life. Further, they found that most cases of significant differences between the three age periods were noted for the comparison of the pre-pubertal to pubertal period. The colour Doppler evaluation score in our study increased gradually and after the 32nd week of life, distinct signals were visible in all the testes. When fertility was achieved in the 36th week of life, in >80% of the testes the course of the vessels was visible within the testicular parenchyma, a fact that might be considered as a possible indicator of fertility.

#### 4.5.5. Correlations between Evaluations

Finally, in order to study potential associations between the various evaluation methods applied in the present study, various correlations were performed. A statistically significant correlation was found between all the evaluation methods, with the exception of the ratio mediastinum testis/testicular parenchyma, which did not show any correlation to any other method. The somatometric evaluations strongly correlated with all other evaluations, confirming the connection of body growth with the onset of puberty. From semen evaluations, total spermatozoa in the ejaculate correlated better than sperm motility in all other clinical and US evaluations. The US evaluation of testicular volume correlated strongly with the clinical evaluation of testicular volume. The grayscale intensity measurements of the various anatomical sections of the testis were also correlated with all other evaluations.

### 4.6. Limitations of the Study

As technology in veterinary imaging modalities advances, improved and more accurate imaging equipment will become available in the future and thus the present results will need to be updated in 5 to 7 years, through the use of equipment and software that will be available to clinicians at that time. That way, results will be updated with the resources of the future, with the aim to improve the accuracies obtained in the present study.

Further, the relatedness among the participating dogs might be considered a further limitation of the study, as the results may not accurately represent the diversity within the broader dog population. The small sample size of the study also presents a limitation in terms of statistical power. Further, confining the study to a single breed might have possibly limited the applicability of the results to other breeds, who may exhibit varying responses due to distinct genetic backgrounds and phenotypic characteristics.

## 5. Conclusions

In Beagle-breed dogs, fertility was achieved in the 36th week of life, when over 200 × 10^6^ spermatozoa were found in the ejaculate. At that age, the animals had reached 95% of weight, 99% of height, 95% of scrotal volume, 75% of testicular volume, and 96% of US testicular volume, compared to respective values in adulthood. The echogenicity of the testicular parenchyma increased with age, from hypoechoic to having medium echogenicity, whilst after the 30th week, it remained stable. The heterogeneity of testis increased significantly with age. The ratio of the grayscale intensity of testicular parenchyma to testis capsule had values < 200 when fertility was achieved. A colour Doppler evaluation of the testicular parenchyma first detected blood flow in the testicular parenchyma in the 22nd week of life. In the 36th week, the course of the vessels was visible inside > 80% of the testes. A significant correlation was found between all the evaluation methods, except for the ratio of grayscale intensity of testicular parenchyma to mediastinum testis. Grayscale intensity values of the testicular parenchyma showed a better association with the total number of spermatozoa in the semen sample collected the same day than with the semen collected two and four weeks afterwards. The use of the Beagle breed might possibly limit the applicability of the results to other breeds, who may exhibit varied responses, due to distinct genetic backgrounds and phenotypic characteristics.

It is feasible that objective measurement of testicular echogenicity and heterogeneity may be useful in a breeding soundness examination of young dogs. The findings of the study may help the diagnostic performance of clinicians on the onset of puberty and fertility in the dog.

## Figures and Tables

**Table 1:** Proportion of testes with each colour Doppler score, at each week of life.

Week	Colour Doppler Score				
	0	1	2	3	4
4 until 20	100%	0%	0%	0%	0%
22	69%	31%	0%	0%	0%
24	31%	19%	44%	6%	0%
26	69%	19%	12%	0%	0%
28	6%	13%	31%	37%	13%
30	0%	6%	25%	44%	25%
32	0%	0%	0%	25%	75%
34	0%	0%	0%	19%	81%
36	0%	0%	0%	19%	81%
38	0%	0%	0%	19%	81%
40	0%	0%	0%	12%	88%

**Table 2:** Results of correlation analysis between the various parameters assessed during the study.

	Weight	Height	Total Sperm	Motility	Scrotum Volume	Testis Volume	US Volume	GI Parench.	GI SD Parench.	GI mt	GI Capsule	Ratio Mt/Parench.
Weight												
Height	0.972											
Total sperm	0.828	0.860										
Motility	0.069	0.762	0.896									
Scrotum volume	0.968	0.995	0.866	0.775								
Testis volume	0.966	0.993	0.866	0.775	0.998							
US volume	0.956	0.984	0.866	0.775	0.988	0.986						
GI parench.	0.840	0.853	0.818	0.685	0.842	0.842	0.885					
GI SD parench.	0.897	0.924	0.866	0.776	0.929	0.928	0.954	0.856				
GI mt	0.957	0.954	0.853	0.747	0.956	0.963	0.975	0.893	0.935			
GI capsule	0.893	0.911	0.832	0.764	0.928	0.919	0.919	0.788	0.935	0.910		
Ratio Mt/parench.	0.470	0.463	0.104	0.062	0.465	0.458	0.451	0.185	0.455	0.461	0.393	
Ratio capsule/parenh.	−0.7501	−0.758	−0.828	−0.733	−0.748	−0.759	−0.780	−0.846	−0.769	−0.804	−0.712	−0.030

(US volume: ultrasonographically evaluated testicular volume; GI parench.: grayscale intensity values of testicular parenchyma; GI SD parench.: standard deviation of grayscale intensity values of testicular parenchyma; GI mt: grayscale intensity values of mediastinum testis; GI capsule: grayscale intensity values of testicular capsule; Ratio Mt/parench.: ratio of grayscale intensity values of mediastinum testis to testicular parenchyma; Ratio capsule/parench.: ratio of grayscale intensity values of testicular capsule to testicular parenchyma).

**Table 3:** Statistical significance (*p*) of results of correlation analysis between various evaluation parameters assessed during the study.

	Weight	Height	Total sperm	Motility	Scrotum Volume	Testis Volume	US Volume	GI Parench.	GI SD Parench.	GI mt	GI Capsule	Ratio Mt/Parench.
Weight												
Height	<0.0001											
Total sperm	<0.0001	<0.0001										
Motility	0.001	0.0002	<0.0001									
Scrotum volume	<0.0001	<0.0001	<0.0001	0.0001								
Testis volume	<0.0001	<0.0001	<0.0001	0.0001	<0.0001							
US volume	<0.0001	<0.0001	<0.0001	0.0001	<0.0001	<0.0001						
GI parench.	<0.0001	<0.0001	<0.0001	0.001	<0.0001	<0.0001	<0.0001					
GI SD parench.	<0.0001	<0.0001	<0.0001	0.0001	<0.0001	<0.0001	<0.0001	<0.0001				
GI mt	<0.0001	<0.0001	<0.0001	0.0002	<0.0001	<0.0001	<0.0001	<0.0001	<0.0001			
GI capsule	<0.0001	<0.0001	<0.0001	0.0001	<0.0001	<0.0001	<0.0001	0.0001	<0.0001	<0.0001		
Ratio Mt/parench.	0.042	0.046	0.67	0.8	0.045	0.049	0.055	0.45	0.05	0.047	0.01	
Ratio capsule/parenh.	0.0002	0.0002	<0.0001	0.0004	0.0002	0.0002	0.0001	< 0.0001	0.0001	<0.0001	0.0006	0.9

(US volume: ultrasonographically evaluated testicular volume; GI parench.: grayscale intensity values of testicular parenchyma; GI SD parench.: standard deviation of grayscale intensity values of testicular parenchyma; GI mt: grayscale intensity values of mediastinum testis; GI capsule: grayscale intensity values of testicular capsule; Ratio Mt/parench.: ratio of grayscale intensity values of mediastinum testis to testicular parenchyma; Ratio capsule/parench.: ratio of grayscale intensity values of testicular capsule to testicular parenchyma).

## Data Availability

The data presented in this study are in the text. The remaining data are available on request from the corresponding author. The data are not publicly available, as they form part of the Ph.D. thesis of the first author, which has not yet been examined, approved, and uploaded in the official depository of Ph.D. theses from Greek universities.

## Supplementary Materials

The following supporting information can be downloaded at: https://www.mdpi.com/article/10.3390/vetsci11060270/s1, Figure S1: Median animal weight (kg) from 4th to 40th week of life (blue arrow: first ejaculate; orange arrow: spermatozoa first seen in ejaculate; green arrow: total sperm count > 200 × 10^6^ in ejaculate), Figure S2: Median animal height (cm) from 4th to 40th week of life (blue arrow: first ejaculate; orange arrow: spermatozoa first seen in ejaculate; green arrow: total sperm count > 200 × 10^6^ in ejaculate), Figure S3: Median scrotal volume (cm^3^) from 4th to 40th week of life (blue arrow: first ejaculate; orange arrow: spermatozoa first seen in ejaculate; green arrow: total sperm count > 200 × 10^6^ in ejaculate), Figure S4: Median ejaculate volume (mL) from 28th to 40th week of life (blue arrow: first ejaculate; orange arrow: spermatozoa first seen in ejaculate; green arrow: total sperm count > 200 × 10^6^ in ejaculate), Figure S5: Median spermatozoa motility (%) from 30th to 40th week of life (orange arrow: spermatozoa first seen in ejaculate; green arrow: total sperm count > 200 × 10^6^ in ejaculate), Table S1: Median, minimum, and maximum values of animal weight (Kg), animal height (cm), and scrotal volume (cm^3^) from 4th to 40th week of life, Table S2: The median, minimum, and maximum of the volume of the ejaculate, the sperm motility, the total number of spermatozoa, and the viability, Table S3: Median, minimum, and maximum values of the ultrasonographically estimated testicular volume (cm^3^), grayscale intensity values of testicular parenchyma, and the standard deviation of the grayscale intensity values of the testicular parenchyma, Table S4: Median, minimum, and maximum values of the grayscale intensity values of mediastinum testis, the grayscale intensity values of the capsule of the tests, the ratio of grayscale intensity of testicular parenchyma to mediastinum testis, and the ratio of grayscale intensity of testicular parenchyma to the capsule of the testis.
