# Supplementary material for: Ultrasonography of Testicular Maturation and Correlation with Body Growth and Semen Evaluation in Beagle Dog Model
Source: Vet Sci. 2024 Jun 14;11(6):270. doi: 10.3390/vetsci11060270 (PMC11209051; doi:10.3390/vetsci11060270)
Supplement: Supplementary file 1 [file vetsci-11-00270-s001.zip › vetsci-2999681-supplementary.pdf]

Supplementary Files

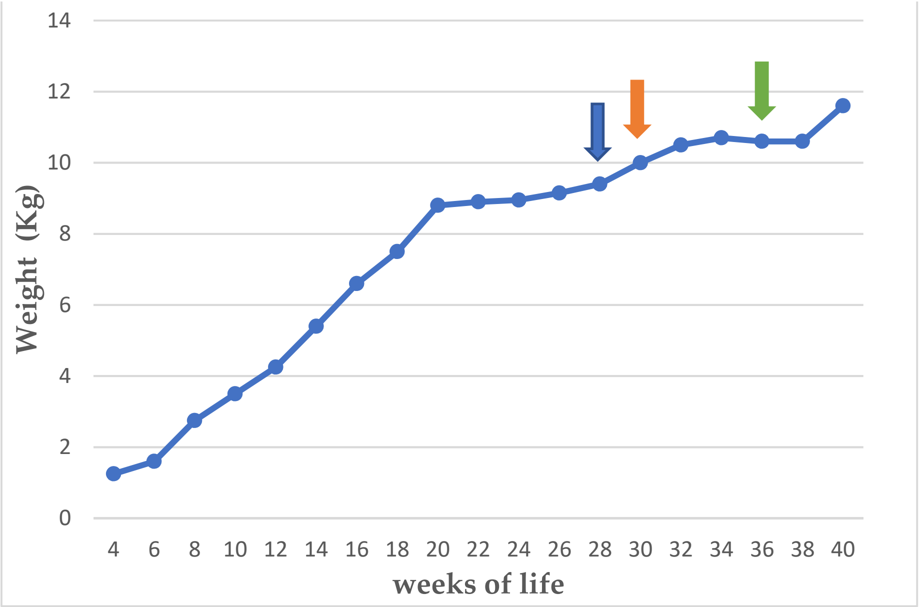

Figure S1. Median animal weight (kg) from 4th to 40th week of life (blue arrow: first ejaculate; orange arrow: spermatozoa first seen in ejaculate; green arrow: total sperm count > 200×10^6 in ejaculate)

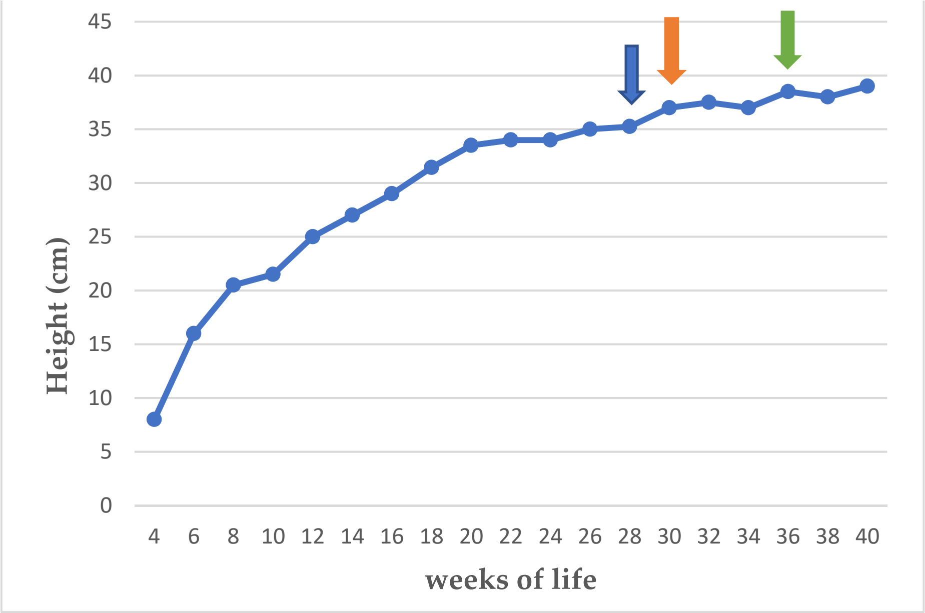

Figure S2. Median animal height (cm) from 4th to 40th week of life (blue arrow: first ejaculate; orange arrow: spermatozoa first seen in ejaculate; green arrow: total sperm count > 200×10^6 in ejaculate).

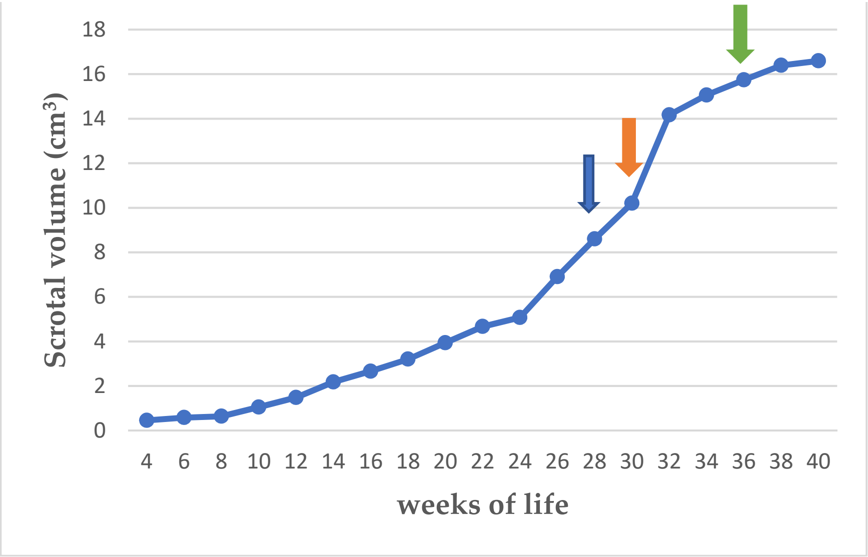

Figure S3. Median scrotal volume (cm^3) from 4th to 40th week of life (blue arrow: first ejaculate; orange arrow: spermatozoa first seen in ejaculate; green arrow: total sperm count > 200×10^6 in ejaculate).

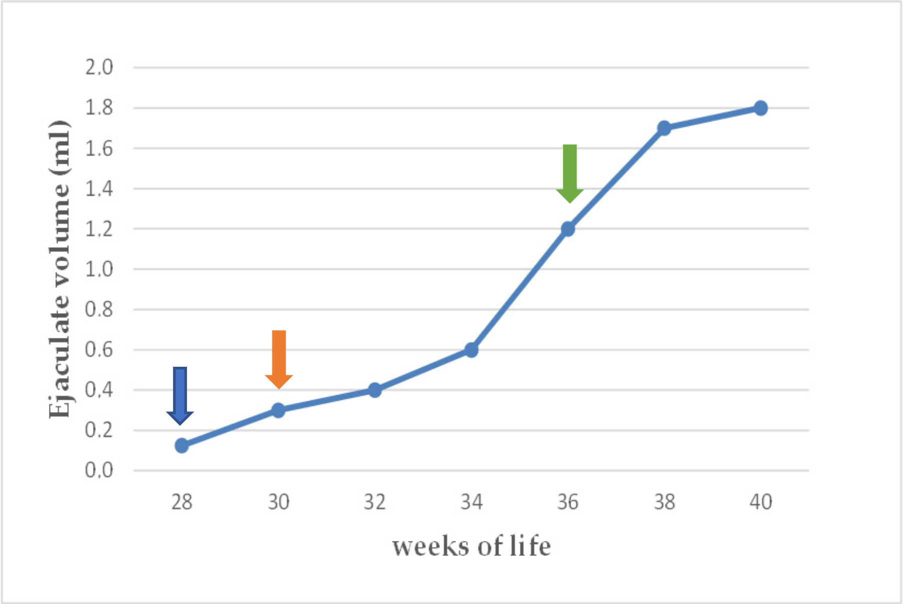

Figure S4. Median ejaculate volume (ml) from 28th to 40th week of life (blue arrow: first ejaculate; orange arrow: spermatozoa first seen in ejaculate; green arrow: total sperm count > 200×10^6 in ejaculate).

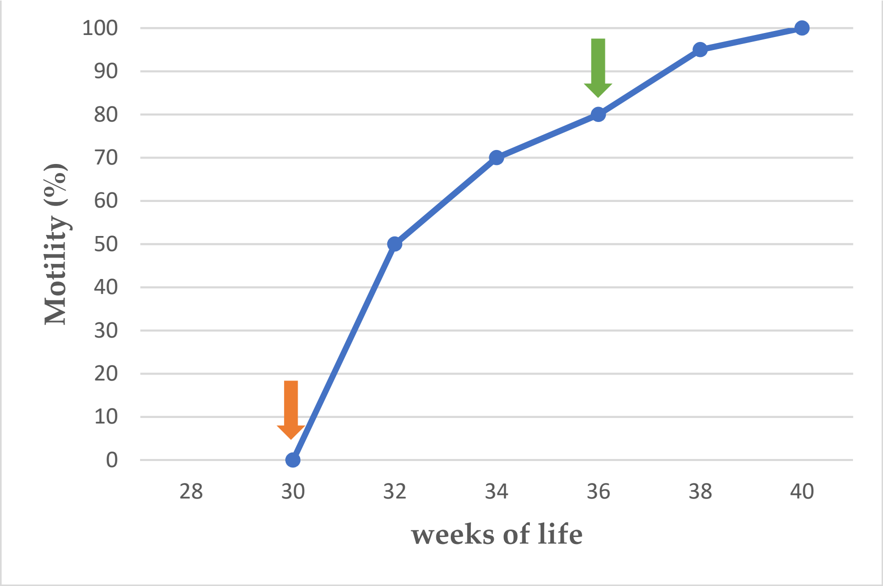

Figure S5. Median spermatozoa motility (%) from 30th to 40th week of life (orange arrow: spermatozoa first seen in ejaculate; green arrow: total sperm count > 200×10^6 in ejaculate).

Table S1. Median, minimum, and maximum values of animal weight (Kg), animal height (cm), scrotal sac volume (cm^3), and testis volume (cm^3) from 4th to 40th week of life.

	Weight (Kg)			Height (cm)			Scrotal sac volume (cm^3)			Testis volume (cm^3)		
Week	Median	Min	Max	Median	Min	Max	Median	Min	Max	Median	Min	Max
4	1.2	1	1.32	8.00	7	13	0.45	0.12	0.45	0.15	0.11	0.16
6	1.6	1.2	1.95	16.00	13	17	0.58	0.16	0.60	0.29	0.18	0.37
8	2.6	2.3	2.9	20.50	18	21	0.64	0.31	1.11	0.51	0.43	0.71
10	3.5	2.9	4	21.50	19.5	24	1.05	0.62	3.18	0.75	0.33	1.19
12	4.3	3.4	4.8	25.00	20.5	27	1.48	0.44	2.57	0.85	0.56	1.80
14	5.8	5	6.8	27.00	22.5	30	2.18	1.03	4.10	1.11	0.21	1.70
16	6.6	5.5	7.3	29.00	26	32	2.66	1.68	6.58	1.20	0.67	1.94
18	7.3	6.1	8	31.45	27.5	36	3.20	2.46	4.42	1.34	0.00	1.65
20	8.0	7.1	9.2	33.50	28	37	3.94	1.83	5.83	1.31	0.86	1.63
22	8.1	7.1	9.1	34.00	31	37	4.67	2.93	10.75	1.86	1.20	2.42
24	8.6	7.9	9.1	34.00	32	39	5.07	3.76	9.40	2.18	1.42	3.35
26	8.9	8.5	9.8	35.00	33	37	6.91	4.76	10.64	2.68	1.98	4.65
28	9.4	8.9	9.6	35.25	34	37	8.60	6.47	16.19	3.29	2.26	6.41
30	10.3	9.5	11.2	37.00	34	38	10.20	9.25	21.02	5.26	3.14	9.59
32	10.7	9.5	11.8	37.50	34	38	14.17	10.36	20.57	6.88	4.94	10.19
34	10.6	9.8	11.6	37.00	35	38	15.06	12.22	20.82	7.21	5.96	10.63
36	11.1	10.5	12.3	38.50	36	39	15.74	15.26	23.56	9.39	8.96	12.17

38	11.5	10.6	12.1	38.00	36	39	16.39	14.75	23.28	9.38	7.64	12.37
40	11.7	11.4	12.1	39.00	36	41	16.60	15.06	23.00	12.37	8.56	13.99

Table S2. The median, minimum, and maximum of the volume of the ejaculate, the sperm motility, the total number of spermatozoa, and the viability.

	Volume (ml)			Motility (%)			Total number of spermatozoa (million)			Viability (%)		
Week	Median	Min	Max	Median	Min	Max	Median	Min	Max	Median	Min	Max
28	0.12	0.07	0.15	0	0	0						
30	0.3	0.08	0.58	0	0	0	1.7	0.2	5.22	80	80	90
32	0.4	0.09	0.95	50	50	80	1.9	0.8	2.22	90	80	100
34	0.35	0.2	1.37	70	60	80	38	12	120	95	80	100
36	0.65	0.4	2.75	80	70	90	201	189	561	99	95	100
38	1.7	1.2	2.1	95	95	100	545	211	732	100	95	100
40	1.8	1.3	2.2	100	95	100	567	202	624	100	95	100

Table S3. Median, minimum, and maximum values of the ultrasonographically estimated testicular volume (cm^3), grayscale intensity values of testicular parenchyma, and the standard deviation of the grayscale intensity values of the testicular parenchyma.

	US estimated testis volume (cm^3)			Grayscale values of testicular parenchyma			Standard deviation of grayscale values of testicular parenchyma		
Week	Median	Min	Max	Median	Min	Max	Median	Min	Max
4	0.21	0.09	0.49	52	35	70	9.3	8.7	11.3
6	0.22	0.10	0.33	30	28	62	7.0	5.8	9.9
8	0.23	0.12	0.32	36	33	69	8.0	4.2	11.7
10	0.28	0.14	0.43	40	29	69	8.6	6.1	10.4
12	0.40	0.17	0.64	36	31	64	9.2	6.8	12.5
14	0.48	0.31	0.75	46	32	67	9.5	6.4	13.3
16	0.58	0.36	0.78	42	33	62	9.5	6.4	11.4
18	0.70	0.39	0.97	50	36	75	9.0	5.5	12.9
20	0.78	0.30	1.16	50	34	68	9.1	5.8	13.5

22	1.16	0.50	1.55	48	48	75	9.8	8.4	15.8
24	1.07	0.54	1.99	49	46	78	10.2	8.3	16.4
26	1.74	1.03	3.76	69	53	92	13.5	9.3	17.5
28	2.61	0.63	4.86	82	61	107	16.8	10.9	17.7
30	4.52	2.28	7.67	103	71	107	17.0	12.3	18.7
32	6.23	3.80	9.92	104	79	127	17.7	14.1	19.1
34	7.20	5.31	10.73	102	94	142	18.1	14.8	18.5
36	8.27	6.23	12.30	105	96	131	19.0	18.6	22.0
38	8.30	6.36	13.17	103	103	129	23.3	19.0	24.8
40	8.62	7.47	14.04	100	102	127	24.5	22.0	24.9

Table S4. Median, minimum, and maximum values of the grayscale intensity values of mediastinum testis, the grayscale intensity values of the capsule of the tests, the ratio of grayscale intensity of testicular parenchyma to mediastinum testis, and the ratio of grayscale intensity of testicular parenchyma to the capsule of the testis.

	Grayscale intensity values of mediastinum testis			Grayscale intensity values of the capsule of the testis			Ratio of grayscale intensity of testicular parenchyma to mediastinum testis			Ratio of grayscale intensity of testicular parenchyma to the capsule of the testis		
Week	Median	Min	Max	Median	Min	Max	Median	Min	Max	Median	Min	Max
4	63	31	88	144	131	216	107	89	120	289	253	414
6	47	30	55	142	105	202	124	100	227	314	268	503
8	58	38	88	158	109	183	108	102	250	312	282	636
10	59	34	89	135	125	195	130	87	199	340	247	659
12	57	35	73	150	96	174	129	102	229	375	229	653
14	74	53	93	150	101	199	172	100	237	316	274	714
16	71	57	94	165	123	173	169	114	244	358	263	757
18	88	55	113	147	132	187	151	128	270	304	231	831
20	90	59	91	162	127	173	158	124	280	347	261	780
22	96	61	115	158	136	189	176	157	305	263	224	532
24	106	72	125	169	144	203	179	169	267	339	289	436
26	126	76	135	184	131	220	161	155	299	273	221	362
28	142	111	159	184	159	201	154	141	236	236	221	328
30	154	137	189	178	167	210	161	155	248	203	198	314
32	155	145	170	214	198	223	152	121	154	172	162	232
34	160	147	181	217	210	233	151	144	172	190	160	224

36	153	141	198	210	182	231	151	125	178	190	161	201
38	184	167	199	221	201	232	161	152	179	191	181	191
40	190	172	191	217	205	241	167	157	174	191	166	195
